# M1 Macrophages Are a Source of IL-1α: A Driver of Progesterone Metabolism and Myometrial Contraction

**DOI:** 10.3390/cells14211692

**Published:** 2025-10-28

**Authors:** Lubna Nadeem, Maxwell Librach, Adam Boros-Rausch, Benjamin Matthews, Eduardo Aguiar-Cabeza, Oksana Shynlova, Stephen James Lye

**Affiliations:** 1 Lunenfeld Tanenbaum Research Institute, Sinai Health System, Toronto, ON M5G 1X5, Canada; 2 Department of Physiology, University of Toronto, Toronto, ON M5S 1A8, Canada; 3 Department of Obstetrics & Gynecology, University of Toronto, Toronto, ON M5G 1E2, Canada

**Keywords:** progesterone withdrawal, labour onset, macrophages, 20alpha hydroxysteroid dehydrogenase, interleukin 1α, myometrium, inflammation

## Abstract

Progesterone (P4) withdrawal is the key trigger for labour onset. Labour is a sterile inflammatory process involving monocyte infiltration, differentiation into M1 or M2 macrophages (Macs) and contributing to the inflammatory milieu in the uterus. Premature leukocyte influx may lead to preterm birth. Inflammatory stimuli induce intracellular progesterone (P4) withdrawal in myometrial cells (MYOs) through activation of P4 metabolizing enzyme 20alpha-hydroxysteroid dehydrogenase (20α-HSD). We hypothesized that (1) the pro-inflammatory M1-Macs induce 20α-HSD in MYO, which causes P4 withdrawal and MYO contractility, and (2) IL-1α produced by M1-Macs mediates the effect of M1-Macs on intracellular P4 withdrawal in MYO. Human myometrial biopsies from term pregnant women in labour (TL) and not in labour (TNL) revealed higher IL-1α in TL, with M1-Macs in TNL expressing more IL-1α than MYO. In vitro study shows (1) higher expression of IL-1α in M1-Macs compared to M2-Macs; (2) treatment of MYO with IL-1α or M1-Macs increased 20α-HSD and contractility; and (3) blockade of IL-1α, AP-1 transcription factors, or co-treatment with non-metabolizable progestin R5020 inhibit these effects. Our findings highlight the role of tissue-resident M1-Macs in regulating intracellular P4 metabolism and suggest that M1-Macs-derived IL-1α may facilitate P4-withdrawal and uterine contractility associated with labour onset.

## 1. Introduction

Preterm birth (PTB) accounts for approximately 9.6% of all pregnancies [1] and is a major contributor to perinatal morbidity and mortality [2,3]. The etiology of PTB is multifactorial: while the uterine infection contributes to 40% of cases, a significant proportion of PTB occur spontaneously without any apparent cause [4]. One of the key factors that maintains pregnancy by promoting uterine quiescence is the steroid hormone progesterone (P4). In most mammals, the decline in systemic P4 levels (“P4 withdrawal”) precedes labour onset. P4 withdrawal leads to activation of uterine smooth muscle (myometrium), resulting in forceful labour contractions and delivery. In rodent models, the administration of the P4 receptor (PR) antagonist mifepristone (aka RU486) induces labour within 24 h [5]. In contrast to other species, plasma progesterone levels remain elevated in humans and higher-order primates prior to the onset of labour [6]. Nevertheless, clinical evidence indicates that treatment with mifepristone also induces labour in pregnant women, suggesting that progesterone is required for the maintenance of pregnancy [7,8,9]. Our previous work has demonstrated that in human (and murine) myometrium, the regulation of P4 levels occurs intracellularly by the enzyme 20alpha-hydroxysteroid dehydrogenase (20α-HSD, encoded by aldo-ketoreductase isoform 1 C1/*AKR1C1*) [10]. The expression of 20α-HSD in the myometrium is induced by inflammatory stimuli (pathologic or physiologic) via Activating Protein-1 (AP-1) and NF-kB-mediated transcription [11], and is elevated in the myometrium before labour onset during term and preterm. 20α-HSD effectively catalyzes the conversion of P4 into its inactive form, 20α-hydroxyprogesterone (20α-OHP), hence reducing intracellular progesterone levels and inhibiting P4 signaling. The intracellular inactivation of P4 leads to the unliganding of PRs, resulting in a “functional” P4 withdrawal [10]. The role of 20α-HSD is further highlighted by findings that the progestin (Promegestone, aka R5020), which is not a substrate of 20α-HSD, effectively inhibits preterm and term labour in mice by maintaining P4/PR signaling in the myometrium [12] and cervix [13]. Therefore, the intracellular inactivation of P4 in myometrial cells is the key step in the initiation of labour onset in human pregnancy.

Term labour is a sterile inflammatory process, governed by a complex inflammatory cascade involving the production of cytokines and chemokines by the uterine tissues and influx of immune cells to the uterus, which further amplify pro-inflammatory signals. This physiological or “sterile” inflammation triggers cervical ripening, fetal membrane weakening, and myometrial contractility, culminating in labour onset. It was noted that term and preterm labour have some common features [14]. For instance, we showed previously that the influx of maternal peripheral immune cells (mainly monocytes) into different uterine compartments (myometrium and decidua) represents a critical step in uterine remodeling and preparation for labour in mice [5,15,16]. Similarly, immune cell infiltration of uterine tissues is central to the infection-induced PTB, as blocking the chemokine receptor CX3CR1 reduced LPS-induced preterm labour in mice [17]. The infiltrated monocytes rapidly differentiate into uterine tissue resident macrophages (Macs), which play a critical role in regulating parturition and postpartum uterine remodeling [15]. The monocyte-derived Macs exhibit remarkable plasticity as they can be polarized into distinct subtypes by local environmental factors. They are typically categorized as classical pro-inflammatory (M1) or homeostatic anti-inflammatory (M2) phenotypes. Importantly, an increased number of M1-Macs are reported in the uterine tissues (including the myometrium) associated with term and preterm labour [18], highlighting their possible role in labour onset. M1-Macs produce pro-inflammatory cytokines, including tumor necrosis factor-alpha (TNFα), interleukin (IL-1)α, IL-1β, IL-6, IL-12, IFN Type I, and chemokines such as CXCL1-3, CXCL-5, and CXCL8-10 [19], while M2-Macs contribute to an anti-inflammatory environment by secreting cytokines such as IL-10, IL-12, CCL17, CCL18, and CCL22 [20,21,22,23]. Therefore, it is plausible to speculate that M1-Macs are a main source of inflammatory signals that activate uterine myocytes (MYOs). Importantly, women undergoing term and preterm labour have elevated levels of cytokines IL-1α and IL-1β in their amniotic fluid [24,25,26] and cervicovaginal fluid [26]. Infection can trigger premature secretion of IL-1β and TNF-α by uterine tissues, which initiates inflammation, myometrial activation and the induction of PTB [27]. In contrast, in the absence of infection, sterile inflammatory signals called “alarmins” (i.e., IL-1α, HMGB1, S100A, etc.) are produced in the uterus, causing spontaneous preterm labour [28]. A recent network analysis revealed the enrichment of IL-1α and HMGB1 in the amniotic fluid of women undergoing preterm labour without the rupture of membranes [29]. Systemic administration of IL-1 [30] as well as intra-amniotic administration of alarmins IL-1α or HMGB1 can induce preterm labour in mice [31]. Understanding the mechanisms of sterile inflammation in the uterus is crucial for developing effective strategies to decrease the risk for spontaneous PTB in women occurring in the absence of infection and preventing neonate mortality and morbidity.

Thus, we hypothesized that during term labour M1-Macs (but not M2-Macs) are a source of elevated levels of IL-1α, which can induce 20α-HSD expression, local P4 withdrawal in human MYO, and increased uterine contractility. Our first aim was to fully characterize the in vivo IL1α expression in term, pregnant human myometrium collected before and during spontaneous term labour. We performed a systemic quantitative immunohistochemical analysis of M1- and M2-Macs and MYO in pregnant human myometrial samples. Secondly, to mimic the relationship between MYO and Macs in the labouring human myometrium, we investigated the impact of the direct interaction between human MYO and M1-Macs on IL1α-induced 20α-HSD expression and MYO contractility in vitro. Furthermore, our study provides compelling evidence that the administration of the progestin, Promegestone (aka R5020), can bypass 20α-HSD activity and inhibit M1-Mac/IL1α-induced myocyte contractility, highlighting its potential as a therapeutic agent for labour prevention.

## 2. Materials and Methods

### 2.1. Ethics Approval

This study was approved by the Mount Sinai Hospital (Toronto, ON, Canada) Research Ethics Board (MSH REB #04-0024E and #12-0007-E). Biopsy samples were obtained from the myometrium of consenting healthy term pregnant women who were either non-labouring (TNL) and undergoing elective caesarian section for breech presentation or repeat section, or labouring (TL) and undergoing an emergency caesarian section due to fetal distress (Supplementary Table S1). The tissue was collected in 50 mL Corning Falcon conical centrifuge tubes, each containing 25 mL of cold HBSS with calcium and magnesium (HBSS+/+), 2.5% HEPES (Wisent Inc., Montreal, QC, Canada) and 1% Penicillin/Streptomycin (P/S) (Lonza, Basel, Switzerland), which were immediately transported to the laboratory. Peripheral blood was collected from third-trimester pregnant women (37–40 weeks’ gestation) into EDTA vacutainer tubes.

### 2.2. Immunohistochemistry of Human Myometrium

Human myometrial biopsies from TNL and TL were fixed in 4% PFA, paraffin-embedded, and sectioned at 5-micron thickness. Sections were baked overnight at 60 °C, deparaffinized in three changes in xylene (5 min each), and rehydrated through a graded ethanol series (100% [×3], 95%, 90%, 80%, 70%, and 50%) for 5 min each. Sections were then incubated in 1× TBS for 15 min. Heat-induced antigen retrieval was performed in 10 mM sodium citrate buffer (pH 6.0) using an Instant Pot pressure cooker at high pressure for 30 min, followed by a 30-min cooling period at room temperature. Slides were washed in TBS (1×) for 5 min and blocked in blocking solution (3% BSA, 5% horse serum in TBS) for 1 h at room temperature in a humidified chamber. Serial sections were co-immunostained with antibodies against IL-1α (Alexa Fluor Cy3), the macrophage marker CD68 (Alexa Fluor 488), and either the M1 macrophage marker iNOS (Alexa Fluor Cy5) or the M2 marker ARG-1 (Alexa Fluor Cy5). Primary antibodies were replaced with corresponding IgGs in the negative control for all tissues. 4′,6-diamidino-2-phenylindole (DAPI) (Sigma-Aldrich, Oakville, ON, Canada) was used for nuclear counterstaining and slides were mounted with vectashield-antifade reagent (Vector Laboratories, Inc., Newark, CA, USA). A complete list of antibodies and reagents used in this study is provided in Table S2. Stained slides were scanned using a Zeiss Axio Scan.Z1 system (Carl Zeiss, Oberkochen, Germany) at 200X magnification.

### 2.3. Primary Myometrial Cell Lines

Primary myometrial cells were isolated from tissue samples using a previously described method [32]. Blood vessels were excised, and the myometrial tissue was cut into approximately 1 mm^3^ pieces using fine scissors and forceps. To remove excess blood, the tissue pieces were washed twice with HBSS +/+ and twice with HBSS −/− supplemented with 2.5% HEPES and 1% penicillin/streptomycin (P/S). The samples were placed in Erlenmeyer flasks and swirled in 20 mL of buffer, with careful aspiration between washes. Tissue samples were then incubated with 5–10 mL of enzymatic digestion solution, comprising 10% fetal bovine serum (FBS, Wisent Inc., Montreal, QC, Canada), 1 mg/mL collagenase II (Sigma-Aldrich, Oakville, ON, Canada), 1 mg/mL bovine serum albumin (BSA) (Wisent Inc., Montreal, QC, Canada), 0.15 mg/mL DNase I (Sigma-Aldrich, Oakville, ON, Canada), and 0.1 mg/mL trypsin inhibitor (Sigma-Aldrich, Oakville, ON, Canada), in a rocking water bath at 37 °C and 2 rpm for 1 h. Following incubation, the digested tissues were pipetted 30 times and filtered through a 70 μm nylon filter to obtain a single-cell suspension in 20 mL of cold HBSS−/− containing 10% FBS and 1 mg/mL BSA. Any undigested tissue was re-incubated with fresh enzymatic solution for another hour at 37 °C. The two cell suspensions were then combined. After centrifugation at 200× *g* for 10 min, the cells were washed with Dulbecco’s Modified Eagle Medium (DMEM, Invitrogen, Carlsbad, CA, USA) containing 10% FBS and 1% P/S and passed through a 23G^¾^ gauge needle. Cells were resuspended in complete growth medium (DMEM, 20% FBS, 1% P/S) and seeded in a 10 cm tissue culture dish. The cultures were maintained in a 20% oxygen atmosphere at 37 °C, and the medium was replaced one day after seeding and every 2–3 days thereafter, according to cell line and growth rate.

### 2.4. Myometrial Cell Treatment Regimen

Primary human myometrial smooth muscle cells (passage 3–8) were seeded on 6 or 12-well plates (Corning, Corning, NY, USA) at densities of 100,000 and 75,000 cells/well, respectively, in DMEM media, supplemented with 10% FBS (Wisent Inc., Montreal, QC, Canada) and 1% P/S (Lonza, Basel, BS, Switzerland). Once cells reached confluence of approximately 80–90%, the growth medium of the cells was replaced with serum-free medium (SFM = DMEM with 1% Insulin-Transferrin-Selenium-Ethanolamine (ITS-X, Invitrogen, Carlsbad, CA, USA) and 1% P/S) for 24 h, and then treated with either IL-1α (1–10,000 pg/mL) or its vehicle (PBS/− with 0.1% BSA in the presence or absence of P4 (100 nM). MYO were pre-treated with IL-1α receptor 1 antagonist (IL1RN, 20 ng/mL) for 30 min and then co-cultured for 24 h with M1-Macs, or a neutralizing antibody against human IL-1α (2–4 µg) was added to the M1-Mac/MYO co-culture for 24 h. Validation of IL-1α inhibition in vitro is shown in Figure S1.

### 2.5. Peripheral Blood-Derived Monocytes

Blood samples were obtained from third-trimester pregnant women (37–40 weeks of gestation). Monocytes were isolated using the RosetteSep Human Total Monocyte Enrichment cocktail (STEMCELL Technologies, Vancouver, BC, Canada) according to the manufacturer’s protocol. Briefly, the RosetteSep cocktail supplemented with EDTA was added to whole blood at a concentration of 50 μL per mL of blood and incubated at room temperature for 20 min. The mixture was then diluted with an equal volume of recommended medium (PBS + 2% FBS) and carefully layered on top of Ficoll (Sigma-Aldrich, Oakville, ON, Canada). They were then centrifuged at 1200 rcf for 20 min. The plasma layer was removed, and the monocyte-enriched cell layer was collected from the plasma interface using a micropipette. The isolated monocytes were washed twice with 15 mL of PBS and then resuspended in RPMI cell culture medium supplemented with 10% FBS (Wisent Inc., Montreal, QC, Canada). The cells were counted and plated at a density of 1 × 10^6^ cells per 10 cm culture dish (Corning, Scarborough, ON, Canada).

### 2.6. In Vitro Differentiation and Polarization of Macrophages

For primary monocyte differentiation and polarization, the method described by Karina et al. [33] was used with slight modifications. Monocytes were allowed to differentiate into macrophages for 10 days in media containing granulocyte-macrophage colony-stimulating factor (GM-CSF, Sigma Aldrich, Oakville, ON, Canada) for M1- and Macrophage colony-stimulating factor (M-CSF) for M2-Macs, at a concentration of 100 ng/mL. The media was refreshed every three days, and the cells were visually monitored for morphological changes. Differentiated macrophages were polarized into M1- with LPS (100 ng/mL), and IFNɣ (25 ng/mL), and into M2-Macs with IL-13 (25 ng/mL,) and IL-4 (25 ng/mL) treatments for 48 h. LPS was purchased from Sigma Aldrich, Oakville, ON, Canada, and all other reagents were obtained from R&D Systems Inc., Minneapolis, MN, USA. Macrophages were harvested using Macrophage Detachment Solution Promocell (Sigma Aldrich, Oakville, ON, Canada), washed with RPMI supplemented with 10% FBS (Wisent Inc., Montreal, QC, Canada), and pelleted by centrifugation at 200× *g* for 5 min.

THP-1 monocytes were differentiated into macrophages according to the protocol outlined by Shiratori et al. [34] with minor modifications. Specifically, THP-1 monocytes were treated with 50 ng/mL of Phorbol 12-myristate 13-acetate (PMA) for 48 h. Following this initial differentiation period, the cells were incubated in complete medium for an additional 48 h. To induce M1 polarization, the macrophages were subsequently exposed to LPS at a concentration of 50 ng/mL and IFN-γ at 20 ng/mL for 48 h. For M2 polarization, the cells were treated with IL-4 at a concentration of 20 ng/mL for 48 h.

### 2.7. Immunocytochemistry (ICC)

Peripheral blood monocytes or THP-1 -derived macrophages were polarized into M1 and M2 phenotypes on 8-well chamber slides using a previously described protocol [34]. Cells were fixed with freshly prepared 4% PFA for 10 min at 37 °C. Cells were then washed with 1X PBS^−/−^ three times, permeabilized with 0.02% Triton-X (Sigma Aldrich, Oakville, ON, Canada) for 5 min, washed 3X, and blocked with 1% BSA in PBS−/− for 60 min. Cells were incubated with a rabbit polyclonal antibody for IL-1α (1:100 dilution) overnight at 4 °C. The following day, slides were washed 3X with PBS^−/−^ and incubated with a donkey anti-rabbit Alexa Fluor 488 secondary antibody (Thermofisher Scientific, Mississauga, ON, Canada, 1:1000 dilution) for 1 h at room temperature. DAPI was used for nuclear staining for 10 min at room temperature. Slides were washed and mounted with antifade mounting medium (Thermofisher Scientific, Mississauga, ON, Canada). Images were taken at 200X magnification on a DMI spinning disc confocal microscope (Leica, Wetzlar, HE, Germany).

### 2.8. Image Analysis

Fluorescence images from human myometrial biopsies were analyzed using QuPath (version 0.6; an open-source software for digital pathology, available at https://qupath.github.io/ (accessed on July 11, 2025)). For each biopsy or tissue section, full image annotations were generated. Cells were detected using the IL1A channel, and cell segmentation was performed accordingly. Channel-specific training classifiers were created to identify macrophages (CD68^+^), M1 macrophages (CD68^+^iNOS^+^), and M2 macrophages (CD68^+^ARG1^+^) based on marker co-expression (summarized in Supplementary Figure S2). IL-1α fluorescence intensity thresholds (minimum and maximum values) were empirically determined and applied uniformly across all samples to ensure consistent quantification. Non-relevant regions, including blood vessels, background, and imaging artifacts, were classified and excluded from the analysis. Mean fluorescence intensity (MFI) values for IL-1α were exported to Microsoft Excel and further analyzed using GraphPad Prism (version 10.3.1). Outliers were identified and excluded using the ROUT method (Q = 1%). The results were cross-verified through a double-blind analysis by two separate researchers.

Fluorescence intensity of IL-1α in peripheral blood monocyte and THP-1-derived macrophages was quantified using ImageJ software: ImageJ 2.16.0/1.54p; Java 1.8.0_322[64 bit], following the protocol described at https://theolb.readthedocs.io/en/latest/imaging/measuring-cell-fluorescence-using-imagej.html (accessed on 1 January 2025). Corrected Total Cell Fluorescence (CTCF) was calculated using the formula:

CTCF = Integrated Density − (Area of selected cell × Mean fluorescence of background).

### 2.9. Protein Extraction

Protein extracts were prepared as previously described [11]. Briefly, cells were washed three times with ice-cold PBS and resuspended in a lysis buffer containing 1 M Tris/HCl (pH 6.8), 10% SDS, 5% glycerol, and 1% Halt protease and phosphatase inhibitor cocktail (Thermo Fisher Scientific, Mississauga, ON, Canada). The lysates were transferred to pre-chilled tubes, vortexed for 15 s, and kept on ice for 10 min. Cell lysates were sonicated at 2.5 dB using an XL-2000 Sonicator (Misonix Inc., Farmingdale, NY, USA) for 10 s, cooled on ice for 5 min, heated at 95 °C for 5 min, cooled on ice for 5 min, and centrifuged at 14,000 rpm for 25 min at 4 °C. Supernatants were collected in pre-chilled 1.5 mL Eppendorf tubes and stored at −20 °C. Protein concentrations were measured using the Pierce BCA Kit (Thermo Fisher Scientific, Mississauga, ON, Canada) according to the manufacturer’s instructions.

### 2.10. Immunoblotting

Western immunoblotting was performed as previously described [11]. Briefly, 20–40 μg of total protein was run on 10–12% TG-SDS-polyacrylamide gels under reducing conditions. BLUelf Prestained Protein Ladder (GeneDireX, Taoyuan, Taiwan) was loaded beside the samples as a reference for protein size. Proteins were transferred to PVDF membranes using Trans-Blot (BioRad, Mississauga, ON, Canada). Membranes were blocked for 1 h at room temperature with 5% milk in TBS-T, followed by overnight incubation with primary antibodies (diluted in blocking solution) at 4 °C. The Next day, the membranes were washed with TBS-T 3X, 10 min each and incubated with HRP-conjugated secondary antibodies (Thermo Fisher Scientific, Mississauga, ON, Canada 1:5000) for 1 h. Following three additional washes with TBS-T, membranes were prepared for chemiluminescent labeling and imaging. Details of all antibodies used in this study are provided in Supplementary Table S2. Band intensity analysis was performed using Image Lab Software:Version 4.0.1 build 6 (Bio-Rad, Mississauga, ON, Canada). The protein targets of interest were normalized to the housekeeping proteins Tubulin or ERK2.

### 2.11. Real-Time PCR

Total RNA was extracted from cultured cells using the RNeasy Mini Kit (Qiagen, Toronto, ON, Canada) according to the manufacturer’s instructions. Following extraction, RNA quality and concentration were assessed using a spectrophotometer, ensuring A260/A280 ratios of ~1.8–2.0. Complementary DNA (cDNA) synthesis was carried out following the manufacturer’s instructions (iScript cDNA synthesis kit, Bio-Rad, Mississauga, ON, Canada) using 1 µg of RNA in a total reaction volume of 20 µL. Quantitative real-time polymerase chain reaction (PCR) was executed utilizing LuminoCt SYBR Green QPCR READYMIX (Sigma Aldrich, Oakville, ON, Canada), a CFX-384 Real-Time System C1000 Thermal Cycler (Bio-Rad, Hercules, CA, USA), and specific primer pairs (Supplementary Table S3). For each PCR reaction, 5 ng of cDNA were utilized, with all reactions conducted in triplicate. The cycle threshold (Ct) values for each sample were recorded. Gene expression levels were normalized to four housekeeping genes (*HPRT*, *SDHA*, *YWHAZ*, *TBP*), and the expression data were processed using CFX Manager software (version 2.1).

### 2.12. Collagen Gel Contraction Assay

The collagen gel contractility assay was adapted from [35]. Confluent primary myometrial cells were harvested with 0.05% trypsin-EDTA, neutralized with culture media, and centrifuged at 200× *g* for 5 min. The cell pellet was resuspended in DMEM supplemented with 20% FBS, counted, and adjusted to the required density. The rat tail collagen type I solution (~3–4.5 mg/mL in 0.1 N HCl) was adjusted to pH 7.2 using 0.1 N NaOH. 150,000 (6-well) or 100,000 (12-well) cells/well (passages 4–6) were mixed into the neutralized collagen, achieving a final collagen concentration of approximately 1.5 mg/mL with 10% FBS. The mixture was carefully transferred to a 6-well (2 mL/well) or 12-well (1 mL/well) culture plate. For co-culture experiments, M1 macrophages derived from peripheral blood from term pregnant women were seeded along with myometrial cells in the ratio of 1:10. Gels were allowed to polymerize at room temperature for 20 min, followed by incubation at 37 °C. After two days of incubation, the gels were detached from the wells, and the medium was replaced with ITS-containing serum-free DMEM media (SFM). Treatments, including LPS (10 ng/mL) and IL-1α (0.1–10 ng/mL), were added to the SFM (as described in figure legends) in the presence of P4 (100 nM) or R5020 (10 nM). The myocytes were pretreated with inhibitors (T5224, 20 µM) or IL1RN (20–40 ng/mL) for 30 min prior to embedding in collagen gels. Four to six technical replicates per condition were prepared, with each experiment repeated at least three times using human primary myometrial cell lines derived from different term pregnant women, with each condition run in quadruplicates. The LPS group represents a positive control, and SFM, a negative control. Images of the floating gels were captured at “time 0” before detachment and then every day for up to 48–96 h and digitized using a ChemiDoc Imaging System (Bio-Rad, Mississauga, ON, Canada). The area of the gels (mm^2^) was measured using Image J software (Version 4.0.1 build 6). Results were expressed as mean gel area (mm^2^) ± the standard error of the mean (SEM) or by calculating the percent of contraction as compared to the “time 0” control wells.

### 2.13. Statistical Analysis

The Shapiro–Wilk test was conducted for all experimental data sets to test normality. For the analysis of two groups, unpaired *t*-tests were performed for normally distributed data. One-way ANOVA with Dunnett’s multiple comparisons post-test was employed to determine the significance between data sets comprised of more than two groups (with normal distribution). A two-way ANOVA with a Dunnett’s multiple comparisons post-test was employed to determine the significance between data sets comprised of multiple groups with two parallel variables (with normal distribution). The Rout or Grubbs’s outliers test was implemented with the assumption of a normally distributed population. Statistical analysis was performed using Prism software-Version 10 (GraphPad Software Inc., CA, USA). The significance level was set as *p* < 0.05 (*), *p* < 0.01 (**), and *p* < 0.001 (***).

## 3. Results

### 3.1. M1 Macrophages Express Elevated Levels of IL-1α Compared to M2 Macrophages

**Figure 1:**
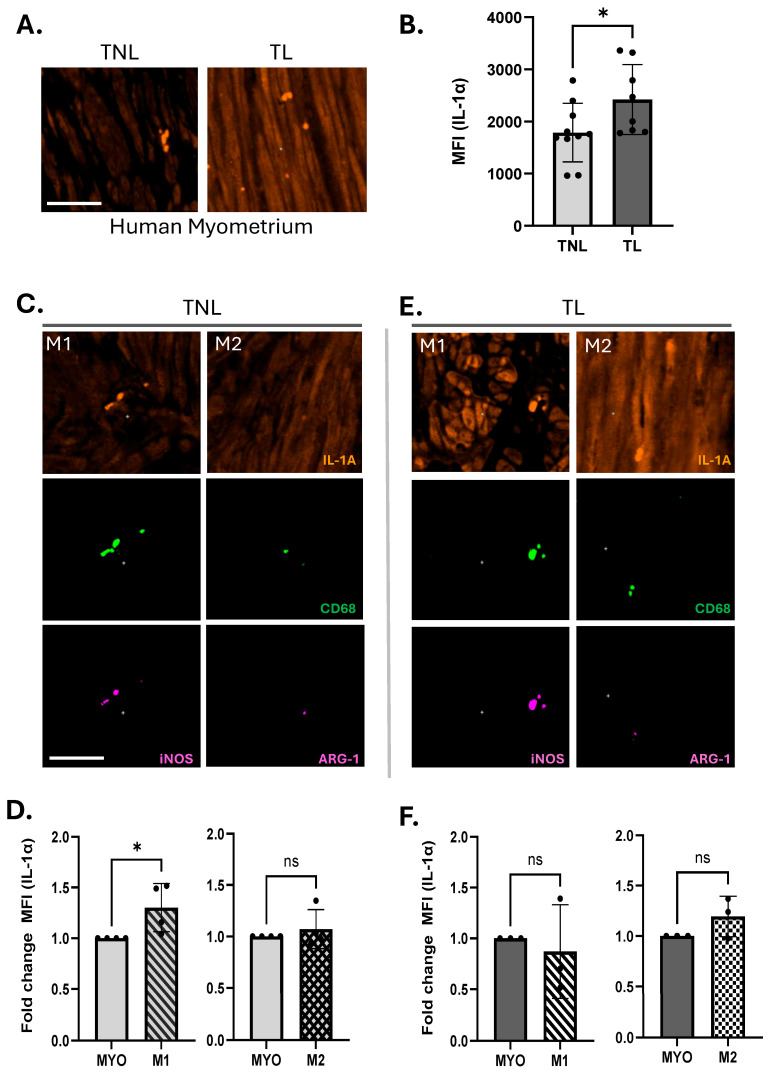
M1 macrophages express elevated levels of IL-1α in non-labouring human myometrium. (**A**) Representative immunofluorescence images depicting IL-1α (orange-red) in myometrial biopsies from women at term not in labour (TNL) and in labour (TL). (**B**) Quantitative QuPath analysis of IL-1α mean fluorescence intensity (MFI) in myometrial myocytes, comparing TNL (*n* = 5) and TL (*n* = 4) samples; data represent 4–5 biological replicates with two technical replicates each. Statistical significance was assessed using unpaired Student’s *t*-test (* *p* ≤ 0.05). Myometrial tissues from TNL and TL women were subjected to immunofluorescence staining with IL-1A (orange-red), pan-macrophage marker CD68 (green), M1 macrophage marker iNOS (pink) or M2 macrophage marker ARG-1 (pink). Representative images (**C**,**E**) Immunofluorescence staining for IL-1α (orange-red), pan-macrophage marker CD68 (green), M1 macrophage marker iNOS (pink), and M2 macrophage marker ARG-1 (pink). Representative images of TNL (**C**,**D**) and TL (**E**,**F**) tissues. (**D**,**F**) Quantitative Qupath analysis of IL-1α MFI in myocytes versus M1 (CD68+ iNOS+) and M2 (CD68+ ARG-1+) macrophages; data are expressed as fold change relative to myocytes. Statistical significance was determined by unpaired Student’s *t*-test, denoted by ‘*’ = *p* ≤ 0.05, ns = non significant. Scale bar = 50 μm.

We first investigated IL-1α levels in term human myometrium obtained from non-labouring (TNL) and labouring (TL) women. Myometrial smooth muscle cells were identified by immunostaining of Smooth Muscle Actin (SMA) (Figure S3), and Macs by a specific marker, CD68 (Figure 1). Given that Macs are known to secrete pro-inflammatory cytokines, we aimed to investigate their role as a source of IL-1α. Positive IL-1α staining was clearly detected in both the MYO and Macs (Figure 1A,B). Immunofluorescent analysis revealed significantly higher levels of IL-1α in the TL myometrium, as compared to TNL (Figure 1A,B).

To identify polarized Macs in vivo in TNL and TL human myometrium, we used M1 (CD68+iNOS+) and M2 (CD68+AGR-1+) markers and confirmed that both sub-types (M1 and M2) express IL-1α. Notably, in the TNL samples, M1-Macs expressed significantly higher levels of IL-1α compared to MYO (Figure 1C,D, left panel), while M2-Macs expressed IL-1α levels comparable to those of MYO (Figure 1C,D, right panel). During TL, uterine MYO, as well as M1- and M2-Macs expressed significantly higher IL-1α levels compared to TNL (Figure 1A,B,E,F and Figure S4).

**Figure 2:**
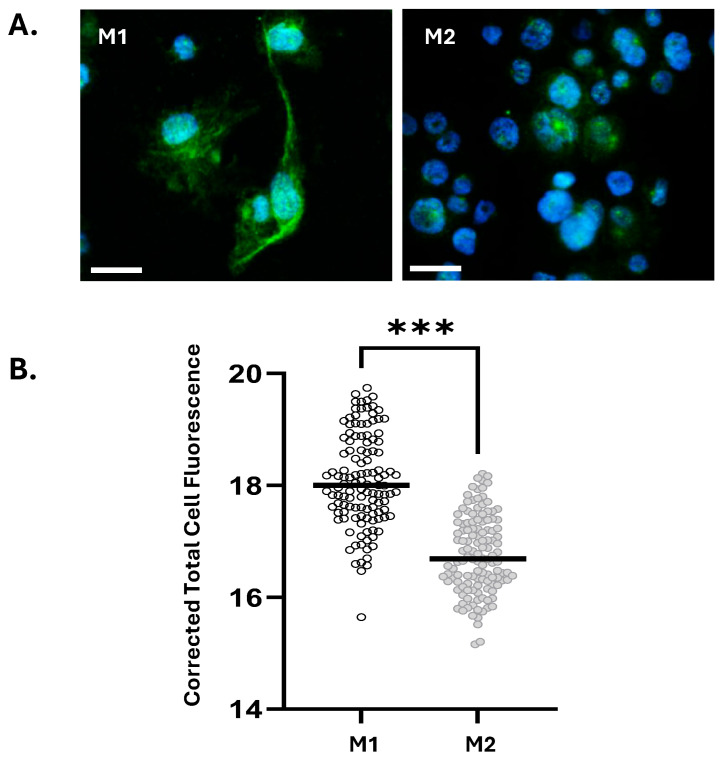
M1 macrophages express elevated levels of IL-1α compared to M2 macrophages. (**A**) Representative images of immunofluorescence staining for IL-1α protein in M1 and M2 macrophages. Scale bar = 32 μm. (**B**) Quantification of immunofluorescence staining for IL-1α protein in M1 and M2 macrophages. Graph shows Corrected Total Cell Fluorescence determined by CTCF analysis using ImageJ software. Data are pooled from three independent experiments. Data was normalized using log2(Y) transformation and statistical significance was determined using unpaired student’s *t*-test and is denoted by ‘***’ = *p* ≤ 0.001.

Next, we examined in vitro IL-1α expression in primary Macs derived from peripheral blood monocytes of term pregnant women. Following polarization to M1 and M2 phenotypes (see Methods), IL-1α was assessed by immunocytochemistry. Analysis showed significantly elevated IL-1α fluorescence levels in M1-Macs compared to M2-Macs (Figure 2A,B). Additionally, we differentiated human monocytic THP-1 cells into Macs and polarized them into M1- and M2-Macs. The levels of *IL1A* transcripts, quantified using real-time PCR, revealed a significant increase in M1-Macs compared to M2-Macs (Figure S5A). Immunostaining for IL-1α protein corroborated these findings, demonstrating a significant increase in IL-1α levels in M1-Macs compared to M2-Macs (Figure S5B). Collectively, our results indicate that M1-Macs serve as a significant source of IL-1α, thereby contributing to sterile inflammation in the uterus.

### 3.2. IL-1α Induces 20a-HSD Levels and Contractility in the Myocytes and This Effect Is Mediated by AP-1 Transcription Factors

**Figure 3:**
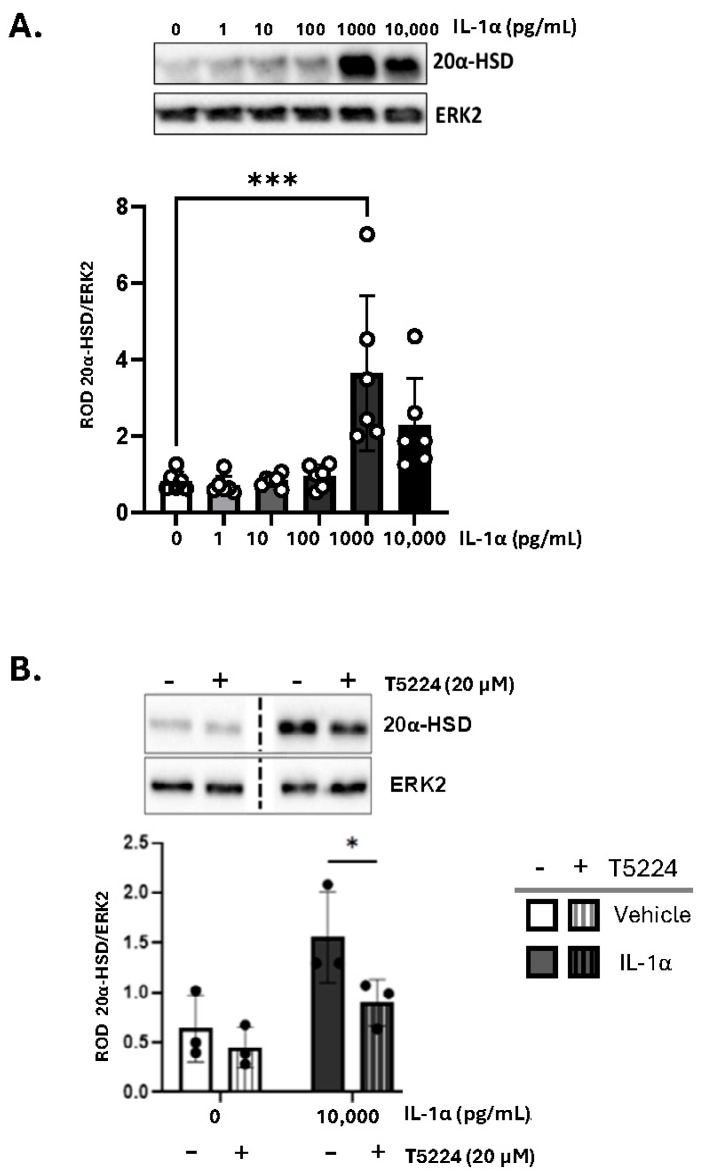
(**A**) IL-1α induces 20α-HSD protein levels in primary human myometrial cells. Representative Western blots and densitometric analysis of 20α-HSD levels in human myometrial cells treated with varying doses of IL-1α (0–10,000 pg/mL) for 24 h. The graph shows 20α-HSD normalized to ERK2 protein (loading control). Data are presented as mean ± SD (*n* = 6). Statistical comparisons were made between the control group (untreated, white bar) and IL-1α-treated groups (grey bars). Statistical significance was determined by One-way ANOVA followed by Dunnett’s multiple comparisons, ‘***’ denotes *p* ≤ 0.001. (**B**) Activator Protein-1 transcription factors block the effect of IL-1α on 20α-HSD levels in primary human myocytes. Representative Western blots and densitometric analysis illustrating the levels of 20α-HSD in myocytes when treated with IL-1α with/without pre-treatment with AP-1 inhibitor; T5224 (20 μM) for 30 min. Bar graphs show data normalized to ERK2. Data presented as mean ± SD (*n* = 3). Statistical comparisons were made using Two-way ANOVA with Šídák’s multiple comparisons test. ‘*’ denotes *p* ≤ 0.05.

Inflammatory stimuli elicited by bacterial endotoxin induce intracellular P4 withdrawal in the myometrium via activation of 20α-HSD [11]. To assess the effect of sterile inflammation (i.e., IL-1α) on 20α-HSD expression, primary human MYO were treated with increasing concentrations of IL-1α for 24 h. A significant increase in 20α-HSD protein expression was detected at 1000 pg/mL of IL-1α (Figure 3A). All experiments were performed in the presence of P4 (100 nM); however, the effect of IL-1α on MYO was independent of hormone treatment (Figure S6).

We reported earlier that the expression of 20α-HSD in myocytes is regulated by the AP-1 family of transcription factors (i.e., cFOS, JUND, etc.) [11]. Since IL-1α is known to activate AP-1 signaling pathway [36], we investigated whether the induction of 20α-HSD in MYOs by IL-1α is mediated by AP-1 signaling. The phosphorylation levels of cFOS protein were assessed in human MYOs cells treated with IL-1α and a significant upregulation of phospho-cFOS was detected after 60 min (Figure S7A). When cultured human MYOs were pre-treated with the selective AP-1 inhibitor (T-5224) for 30 min, the induction of 20α-HSD protein levels by IL-1α was significantly reduced (Figure 3B). Validation of T-5224 is shown in Figure S7B.

**Figure 4:**
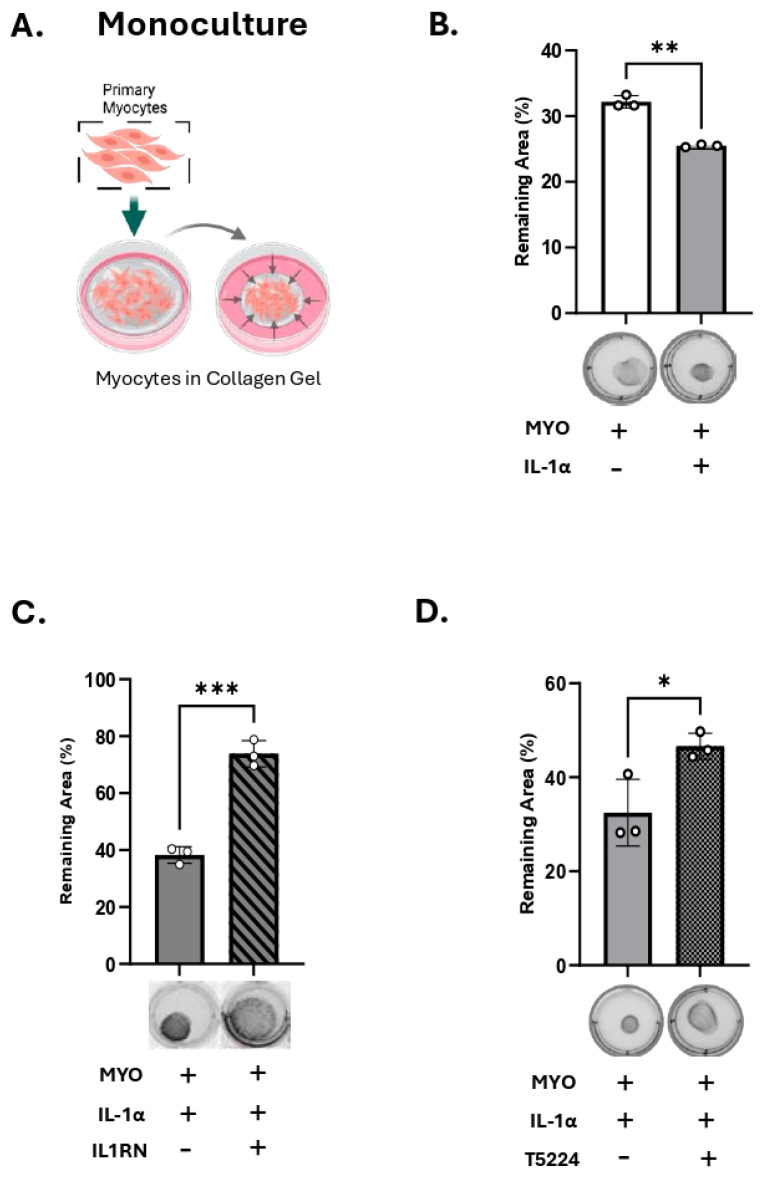
IL-1α induces myocyte contractility. (**A**) Schematic (created with BioRender.com), of primary human myocytes embedded in collagen gel (monoculture). (**B**–**D**) Representative images and area quantification of collagen lattices containing primary human myometrial cells treated with IL-1α (100 pg/mL), with/without IL1R1 receptor antagonist; IL1RN (40 ng/mL), or AP-1 inhibitor; T5224 (20 μM) in the presence of progesterone (P4,100 nM). Graphs indicate the contraction of collagen lattices after 48 h of stimulation, presented by percent area remaining at 48 h compared to 0 h. Data presented as mean ± SD (*n* = 3). Statistical difference was determined using Student’s *t*-test, denoted by asterisks ‘*’ = *p* ≤ 0.05; ‘**’ = *p* ≤ 0.01; ‘***’ = *p* ≤ 0.001.

We next determined the functional consequence of IL-1α treatment on human myocyte contractility using a three-dimensional collagen gel contraction assay. Human MYOs were embedded in collagen gels (monoculture, Figure 4A) and treated with IL-1α or vehicle for 48 h in the presence of P4 (100 nM). We recorded a significant contraction of collagen lattices following IL-1α treatment compared to vehicle-treated control gels (white vs. grey bars, Figure 4B). When MYO were pre-treated with IL-1R1 antagonist (Figure 4C, IL1RN, grey versus checkered bar), and then embedded in gels, the effect of exogenous IL-1α on MYO contraction was significantly attenuated. Following treatment with IL-1α, we assessed myometrial cell viability, which indicated no toxic effects of the cytokine on MYO survival after 48 h (Figure S7C). In addition, when MYO were pretreated with the AP-1 inhibitor T5224 (Figure 4D, grey versus diagonal lines bar), the effect of IL-1α on MYO contractility was blocked. These results suggest that IL-1α-induced myocyte contractility is mediated by AP-1 transcription factors.

### 3.3. M1-Macrophages Induce 20α-HSD Protein Expression and Contractility of Human MYO

**Figure 5:**
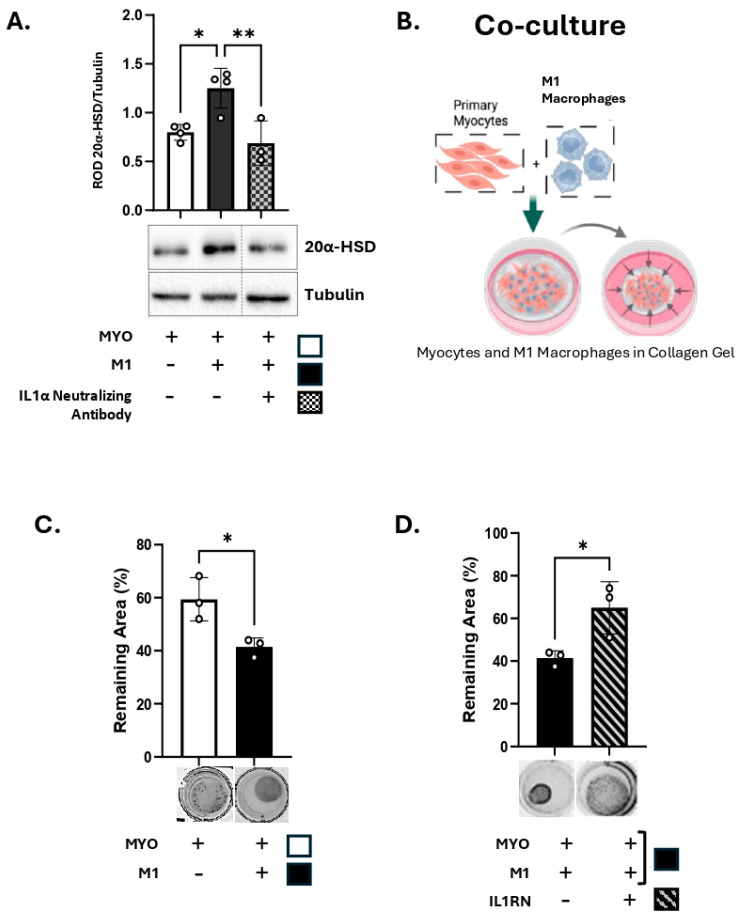
M1 macrophages induces 20α-HSD and myocyte contractility through IL-1α. (**A**) Representative Western blots and densitometric analysis illustrating 20α-HSD levels in primary human myocytes (MYOs) in monoculture (white bar) or co-cultured with M1 macrophages (M1-Mac) (black bar), with/without inhibition of IL-1α signalling by IL-1α neutralizing antibody (4 μg, grey bar with checkerboard pattern) in the presence of P4 (100 nM). The graph represents mean relative 20α-HSD levels relative to Tubulin (loading control). Data are shown as mean ± SD (*n* = 4). Statistical analyses were performed using One-way ANOVA followed by Dunnett’s test of multiple comparisons. Statistical significance is denoted by asterisks: ‘*’ = *p* ≤ 0.05, ‘**’ = *p* ≤ 0.01. (**B**) Schematic (created with BioRender.com), of collagen gel contractility of M1-Mac and MYO in co-culture. (**C**,**D**) Representative images and quantification of collagen lattices embedded with M1-Mac:MYO in a ratio of 1:10 and treated with/without IL1R1 antagonist (40 ng/mL). Collagen gels were incubated for 48 h in the presence of progesterone (100 nM). Graphs show the contraction of collagen lattices represented by percent area remaining at 48 h compared to 0 h. Data represents mean ± SD (*n* = 3). Statistical analyses were performed unpaired *t*-test. Statistical significance is denoted by asterisks: ‘*’ = *p* ≤ 0.05.

We further investigated the effect of IL-1α produced by M1-Macs on the myometrial 20α-HSD levels. Primary human MYO were cultured in vitro either alone or in co-culture with M1-Macs (Macs to MYO ratio 1:4), and 20α-HSD protein levels were examined by immunoblotting. Our results show that, similar to the treatment of human MYO with exogenous IL-1α (Figure 3A), co-culture of MYO with M1-Macs induces 20α-HSD protein expression as compared to MYO monoculture (white vs. black bars, Figure 5A). Importantly, the inhibition of IL-1α signaling in the co-culture of M1-Mac/MYO by neutralizing antibody against human IL-1α prevented the induction of 20α-HSD by M1-Macs (patterned bars, Figure 5A). Validation of IL-1α inhibition by IL-1α neutralizing antibody was performed by immunoblotting (Figure S1).

When human MYOs were embedded in 3D collagen gels with M1-Macs (M1-Mac:MYO co-culture, 1:10) in the presence of P4 (100 nM), M1-Macs induced MYO contractility to levels similar to that of MYO monoculture treated with IL-1α (Figure 5B,C). When MYOs were pretreated with IL1RN before embedding in collagen gel, the M1-Macs-induced MYO contractility was significantly reduced (Figure 5D). We concluded that the MYO contractility induced by M1-Macs is at least partially mediated via IL-1 signalling.

### 3.4. R5020 Blocks the Effect of IL-1α and M1-Macrophages on Myocyte Contractility

**Figure 6:**
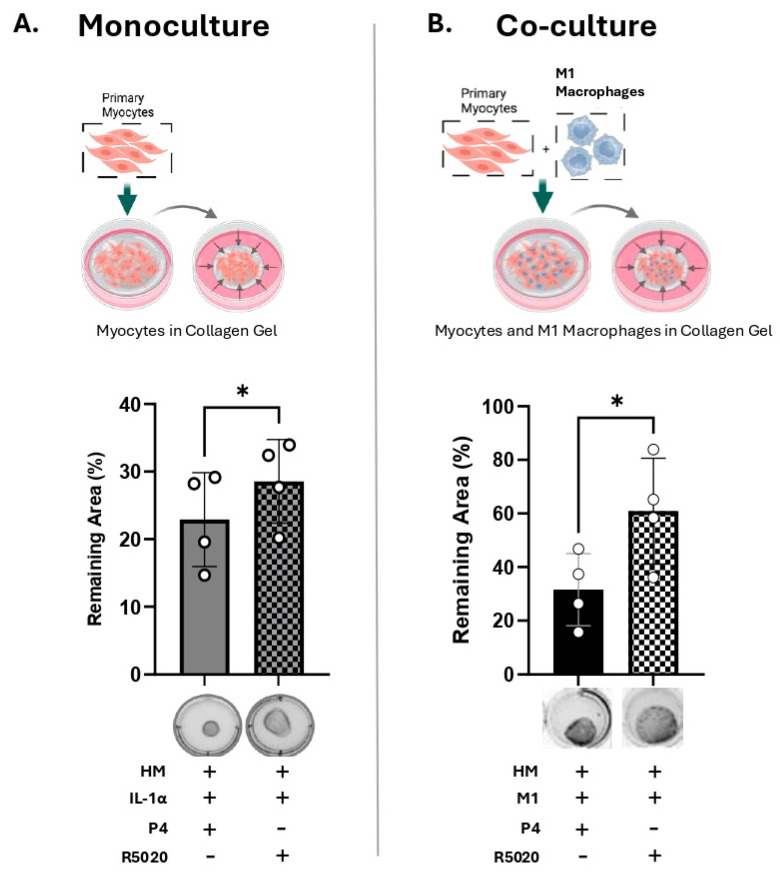
R5020 blocks the effect of IL-1α and M1-Macrophages on myocyte contractility. Schematic (created with BioRender.com), representative images and quantification of collagen lattices embedded with (**A**) primary human myometrial cells (MYOs) alone (monoculture) treated with IL-1α (100 pg/mL), with progesterone (P4, 100 nM) or promegestone (R5020, 10 nM); (**B**) MYO co-cultured with M1 polarized macrophages (M1-Macs) in a ratio of 1:10 (M1-Macs:MYO) with P4 (100 nM) or R5020 (10 nM). Graphs show the contraction of collagen lattices represented by percent area remaining at 96 h compared to 0 h. Data represents mean ± SD (*n* = 4). Statistical analyses were performed using unpaired *t*-test. Statistical significance is denoted by asterisks: ‘*’ = *p* ≤ 0.05.

Next, we determined whether the progestin R5020 (non-metabolizable by 20α-HSD) can inhibit IL-1α-induced MYO contractility. Primary MYO embedded within collagen gels (monoculture) were treated with IL-1α (100 pg/mL) in the presence of P4 (100 nM) or R5020 (10 nM). We found that in contrast to P4, R5020 supplementation significantly inhibited IL-1α-induced collagen gel contraction (grey versus checkered bars, Figure 6A). Similarly, in the co-culture model (M1-Macs: MYO, 1:10), MYO contractility induced by M1-Macs was significantly lower in the presence of R5020 compared to P4 (black versus checkered bars, Figure 6B). Thus, we concluded that IL-1α produced by M1-Macs was able to induce 20α-HSD in MYO, which caused P4 metabolism and thus increased MYO contractility, while R5020, which is not metabolized by 20α-HSD, prevented MYO gel contraction.

## 4. Discussion

Immune cells play a crucial regulatory role throughout all stages of pregnancy. Uterine Macs participate in various processes, including implantation, decidualization, placentation, parturition, and postpartum tissue involution [5,15,16,37,38]. As key effectors of the maternal innate immune system, tissue Macs adapt their functions based on signals from their microenvironment [39]. At the maternal–fetal interface, macrophages dynamically shift between M1 and M2 phenotypes throughout pregnancy. M1-Macs are primarily involved in host defense against pathogens, whereas M2-Macs play a role in resolving inflammation and promoting tissue repair [20].

Mechanical stretch of the pregnant myometrium prior to and during labour stimulates the production of cytokines and chemokines from MYO, which mediate uterine inflammation, recruitment and activation of peripheral monocytes, which differentiate into Macs [32,40]. An increased number of M1-Macs have been reported in uterine tissues in vivo during both term and preterm labour [18], suggesting that the inflammatory milieu of the uterus polarizes the infiltrated Macs towards the M1 phenotype. Disruptions in the M1/M2 balance towards excessive M1-Macs and insufficient M2-Macs have been linked to pregnancy complications, including spontaneous abortion [41,42], preterm labour [18,43,44,45], and gestational diabetes mellitus (GDM) [46,47,48,49,50]. Increased number of Macs in the myometrium during term, and spontaneous preterm labour has been associated with increases in cytokine levels, including IL-1α and IL-1β, in the maternal, fetal tissues and cervico-vaginal fluid [19,20,21,22,23,26,32,51]. Thus, we hypothesized that in the absence of infection, in vivo M1-Macs are a significant source of IL-1α in the term human uterus, which can induce local P4 withdrawal in myometrium.

The IL-1 family of cytokines, especially IL-1β and IL-1α, plays a critical role in the pathogenesis of various inflammatory disorders, including inflammatory bowel disease (IBD), atherosclerosis, ischemia/reperfusion injury, coronary artery disease, and PTB. IL-1 cytokines are primarily secreted into the extracellular space, where they act through autocrine and paracrine signaling by binding to the IL-1 receptor (IL1R) [52]. These cytokines exert strong pro-inflammatory effects, which are counteracted in vivo by their natural inhibitor, the IL-1 receptor antagonist (IL1RN, or IL1RA). Under homeostatic conditions, IL-1α is localized intracellularly and is constitutively produced by various cell types, including epithelial, endothelial, stromal, and immune cells, with Macs being a primary source. Expression of IL-1α is amplified by autoregulation [53] and can be induced by Toll-like receptor agonists, pro-inflammatory cytokines (TNF-α and IL-1β), oxidative stress [54,55], fatty acid-induced mitochondrial uncoupling [56], and estradiol [57]. In uterine tissues, IL-1α expression can be induced by redox system imbalances at the maternal–fetal interface [58,59] or increased estradiol levels [60,61]. Importantly, IL-1α administration alone, systemic [24,30] or intra-amniotic [31] is sufficient to induce PTB in mice through activation of the NLRP3 inflammasome.

IL-1α primarily acts as an “alarmin” which is rapidly released upon significant cell damage to trigger early host defense mechanisms [58,62]. However, unlike other members of the family, IL-1α is biologically active in its pro-form, particularly in Macs where the pro-IL-1α acquires membrane localization [63,64]. The membrane-associated pro-IL-1α can signal in a juxtacrine manner by interacting with IL-1R on adjacent cells, thereby initiating localized and potentially focal inflammatory responses [58,63,65]. This property of Macs highlights the potential impact of increased M1-Macs on IL1a signaling in the surrounding MYO. Experimental evidence from murine and primate animal models has shown that both IL-1α and IL-1β are potent inducers of preterm labour [24,31]. IL-1α, however, shows higher efficacy at significantly lower concentrations compared to IL-1β, implying that minimal exposure to IL-1α is sufficient to trigger labour. The biological activity of IL-1α in its precursor form and its membrane presentation in Macs [64] adds to the efficacy of IL-1α over IL-1β, whose activation relies on the inflammasome activation and the cleavage by caspase-1. The increased number of M1-Macs preceding labour may therefore be a primary driver of IL-1α signaling in MYO.

A recent study [66] demonstrated that IL-1β produced by the LPS-activated Macs (M1-Macs) promote in vitro myocyte differentiation and contractility. However, they also showed that IL-1β alone is insufficient to induce myocyte contractility, and that the physical presence of Macs is crucial for connexin 43 (Cx43)-mediated myocyte connectivity/synchronization and contraction. Our study provides the missing pieces of this puzzle and demonstrates that IL-1α from M1-Macs mediates the inactivation of P4, which is necessary for MYO activation and contractility. Since P4 is a primary repressor of Cx43 transcription and trafficking in the MYOs [10], its inactivation via IL-1α-driven P4 metabolism leads to the upregulation of Cx43 and other contraction-associated genes repressed by P4. Furthermore, we propose that the inflammasome activation and subsequent IL-1β signaling are the downstream events of IL-1α signaling, since IL-1α alone is adequate to induce myocyte contraction. IL-1α sets the stage by inducing P4 metabolism, while IL-1β propagates the inflammatory cascade that promotes myometrial differentiation and ultimately facilitates labour onset.

Mechanistically, the induction of 20α-HSD expression by IL-1α was mediated by AP-1 transcription factors. Our previous studies demonstrated that two transcription factors (i.e., AP-1 and NF-κB) play a role in the inflammation-induced expression of 20α-HSD in human myocytes, which contributes to intracellular P4 withdrawal [11]. Additionally, it has been reported that inflammatory conditions can shift the ratio of PR isoforms in favor of PR-A [67]. The dominance of PR-A and activation of AP-1 together contribute to myocyte activation, since the interaction of PR-A with AP-1 heterodimers promotes the transcription of contraction-associated genes such as *GJA-1* (Cx43) [10], which is crucial for myocyte activation and contractility [68]. Collectively, these results highlight the fundamental role of AP-1 as a transcriptional mediator of pro-labour and pro-contractility genes in myometrial cells, including 20α-HSD, effectively bridging inflammation-induced P4 withdrawal and myocyte contractility.

We recently demonstrated that pathogenic stimuli, such as LPS, can induce 20α-HSD expression in human myometrial cells, leading to intracellular P4 withdrawal [11]. Our present data further indicate that both infectious (LPS) and sterile (IL-1α) inflammation similarly upregulate 20α-HSD expression in human MYO, which enhances P4 metabolism and its intracellular depletion—a key step in increasing myometrial contractility and triggering labour.

Importantly, our recent animal studies showed that administration of systemic or intrauterine LPS causes PTL in pregnant mice and induces myometrial expression of 20α-HSD, while the prophylactic administration of R5020 (aka Promegestone) prevented PTL, inhibited inflammation, myometrium contractility, and cervical remodeling [12,13]. R5020 belongs to a group of Selective Progesterone Receptor Modulators (SPRMs). It has been used in post-menopause hormonal replacement therapy and in the treatment of gynecological conditions caused by luteal insufficiency, including premenopausal disorders, and dysmenorrhea [69,70,71]. R5020 can replace P4 in ovariectomized pregnant mice, maintaining pregnancy until full term [72]. In contrast to the progestin 17αOHP, R5020 administration can prevent preterm labour in mice [73]. It possesses a potent progestogenic activity and, (1) binds to PRs with an affinity and specificity higher than endogenous P4 [74,75,76], (2) has a longer half-life in vivo [74,75], and most importantly, and (3) is not a substrate of 20α-HSD. Hence, it can bypass the intracellular metabolism of progesterone by 20α-HSD. In humans, R5020 is metabolized by 21-hydroxylation to trimegestone, which has an even higher affinity and specificity to PRs than R5020 [74,75].

Our present data demonstrate that in vitro supplementation with R5020 effectively inhibited human myocyte contractility induced by IL-1α or stimulated by M1-Macs, as measured by functional gel contraction assay. Notably, this inhibitory effect was more pronounced than that observed with P4 supplementation. IL-1α treatment of MYO in monoculture, as well as M1-Mac interaction with MYO in co-culture, induced MYO contraction. This effect was blocked by R5020, linking it to 20α-HSD-mediated P4 depletion and loss of P4 action. Thus, we speculate that intracellular P4 withdrawal is the key mechanism driving M1-Mac/IL-1α-induced myocyte contractility. Previous human studies have shown that blocking P4 signaling with the PR antagonist Mifepristone induces inflammation, increases myometrial contractility, and promotes cervical ripening [73,77,78], ultimately leading to pregnancy termination [79,80]. Based on our current findings, we propose that maintaining P4 signaling with R5020 could offer an effective progestational therapy for preventing PTB in high-risk pregnancies.

**Figure 7:**
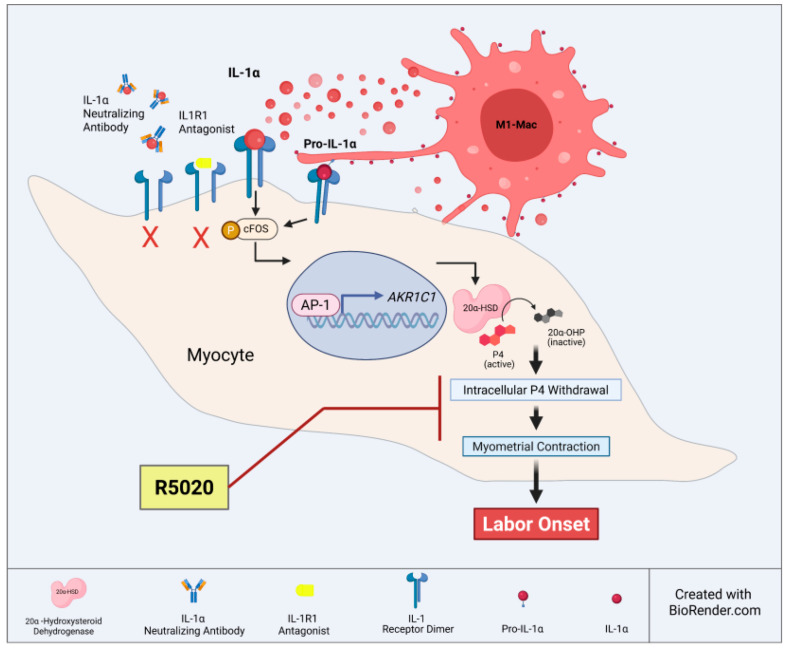
Proposed model of M1 macrophage-mediated intracellular progesterone withdrawal and myocyte contractility. This illustration depicts the role of pro-inflammatory M1 macrophages within the uterine environment as a significant source of cytokine interleukin 1 alpha (IL-1α), which acts via its IL1 receptor 1 (IL1R1).IL-1αsignaling promotes the upregulation of 20α-hydroxysteroid dehydrogenase (20α-HSD, encoded by *AKR1C1* gene) protein levels in myocytes. Inflammation-induced 20α-HSD activity facilitates the metabolism of progesterone (P4) to its inactive analog 20α-OHP, leading to intracellular P4 withdrawal in myocytes. IL-1α-induced P4 withdrawal triggers myocyte activation and increased contractility, ultimately contributing to the onset of labour. Non-metabolizable progestin R5020 (aka Promegestone) can block myocyte contractility and labour onset. Illustration was created with BioRender.com.

In summary (Figure 7), our findings provide deeper insight into the role of M1-Macs as a source of local sterile inflammation in the myometrium, P4 withdrawal, and myometrial activation. The observed upregulation of 20α-HSD in response to IL-1α administration in vitro, which can be replicated by the presence of activated M1-Macs, directly confirms their involvement in these processes. Additionally, our study suggests that premature immune cell activation may function as a trigger for spontaneous preterm labour, even in the absence of infection. Importantly, we showed that R5020 (the stable progestin) alone can counteract the effects of M1-Macs and inhibit MYO contractility. These findings highlight the therapeutic potential of non-metabolizable progestins for preventing inflammation-induced PTB.

## 5. Study Limitations

We acknowledge several limitations in our study. First, our research utilized myometrial biopsy samples and peripheral blood monocytes from term pregnant women. Analyzing myometrial tissue from women experiencing spontaneous, non-infectious PTL, if accessible, would provide valuable comparative insights and help confirm the role of M1-Macs and sterile inflammatory mediators in triggering P4 withdrawal and premature myometrial contractions.

Second, most of our experiments were conducted in vitro using primary myometrial cells derived from human uterine biopsies. While these models are crucial for elucidating cellular mechanisms of labour induction, they may not fully replicate the complex in vivo environment of the human uterus. In addition to M1-Macs, multiple cell types likely contribute to the inflammatory milieu of the pregnant uterus. Conducting in vivo animal studies that specifically target or deplete M1-Macs could provide deeper insights into their unique role in labour onset.

Third, we recognize that IL-1α is not the only pro-inflammatory cytokine produced or released by M1-Macs. Other cytokines, such as IL-1β and IL-6, may also play critical roles in myometrial contractility [81,82], as their expression is associated with both TL and PTL. Future studies examining the contribution of these cytokines to M1-Mac-mediated P4 withdrawal in the myometrium are essential.

## 6. Conclusions

In conclusion, our study demonstrates that pro-inflammatory M1-Macs contribute to myometrial activation during the onset of labour. IL-1α, derived from M1-Macs, plays a key role in facilitating intracellular P4 withdrawal, thereby enhancing myocyte contractility. The non-metabolizable progestin R5020 effectively counteracts M1-Mac-mediated effects and reduces myocyte contractility, highlighting its potential as a therapeutic approach to prevent premature uterine contractions (Figure 7). These findings advance our understanding of the molecular mechanisms driving human labour and highlight potential targets for future interventions to reduce the risk of PTB in high-risk women.

## Data Availability

The original contributions presented in this study are included in the article/Supplementary Materials. Further inquiries can be directed to the corresponding authors.

## Supplementary Materials

The following supporting information can be downloaded at: https://www.mdpi.com/article/10.3390/cells14211692/s1, Figure S1: Validation of IL-1α signaling inhibitors; Figure S2: Detailed workflow of detection and classification of macrophages and myocytes in human myometrium using QuPath analysis. Figure S3: Representative immunofluorescence images of human myometrium from term not in labour (TNL) and term labour (TL), stained with IL-1α, Smooth Muscle Actin staining, and IgG negative controls; Figure S4: Comparative analysis of IL-1A expression in M1 and M2 macrophages versus myocytes within myometrial biopsies from women with term not in labour (TNL) and term labour (TL), as summarized in Figure 1; Figure S5: Analysis of IL1α levels in M1 versus M2 macrophages; Figure S6: Effect of progesterone on M1-Macrophage induced IL1α in myocytes; Figure S7: Validation of AP-1 inhibition by a specific inhibitor; Table S1: Demographics of human myometrium sample donors. Table S2: List of antibodies and their sources used in this study; Table S3: List of primers used in this study for qPCR analyses.
