# Supplementary material for: M1 Macrophages Are a Source of IL-1α: A Driver of Progesterone Metabolism and Myometrial Contraction
Source: Cells. 2025 Oct 28;14(21):1692. doi: 10.3390/cells14211692 (PMC12608973; doi:10.3390/cells14211692)
Supplement: Supplementary file 1 [file cells-14-01692-s001.zip › cells-3934267-supplementary.pdf]

Table S1: Demographics of human myometrium sample donors

Sample ID	Gestational age (weeks + days)	Parity (# of deliveries)	Fetal Sex M/F
TNL #1	39.1	P1	M
TNL #2	38.5	P2	F
TNL #3	37.6	P1	NA
TNL #4	38.3	P1	NA
TNL #5	39	P2	F
TNL #6	38	P1	M
TL #1	38.1	P1	NA
TL #2	40.3	P1	M
TL #3	40.1	NA	M
TL #4	37.7	NA	M
TL #5	40	P1	M

TNL = Term non labouring; TL = Term Labouring; NA = Not available; M = Male; F = Female

Table S2: List of antibodies and their sources used in this study

Name	Specificity	Company	Catalogue #
AKR1C1/20αHSD	Rabbit Polyclonal	GeneTex	GTX105620
Phospho-cFOS (Ser 32)	Rabbit monoclonal	Cell Signaling	5348S
cFOS	Rabbit monoclonal	Cell Signaling	2250S
Phospho-NF-κB p65 (Ser 536)	Rabbit monoclonal	Cell Signaling	3033S
NF-κB p65	Rabbit monoclonal	Cell Signaling	8242S
ERK2	Rabbit Polyclonal	Abcam	Ab227134
Tubulin	Mouse monoclonal	Sigma	T5168
IL-1α	Rabbit Polyclonal	Cell Signaling	84618
Human IL-1α Neutralizing antibody	Mouse monoclonal	Invivogen	mabg-hil1a-3
IL-1α	Rabbit Polyclonal	Cell Signaling	84618
iNOS	Rabbit monoclonal	Abcam	Ab115819
Arginase 1	Rabbit monoclonal	Abcam	Ab133543

Table S3: List of primers used in this study.

Gene Symbol	Forward Primer	Reverse Primer	Accession Number
IL1A	5′-TGGTAGTAGCAACCAACGGGA-3′	5′-ACTTTGATTGAGGGCGTCATTC-3′	NM_000575.5
YWHAZ	5′-ACTTTTGGTACATTGTGGCTTCAA-3′	5′-CCGCCAGGACAAACCAGTAT-3′	NM_001135699.2
SDHA	5′-TGGGAACAAGAGGGCATCTG-3′	5′-CCACCACTGCATCAAATTCATG-3′	NM_004168.4
TBP	5′-CCACAGCTCTTCCACTCACA-3′	5′-CTGCGGTACAATCCCAGAAC-3′	NM_003194.4

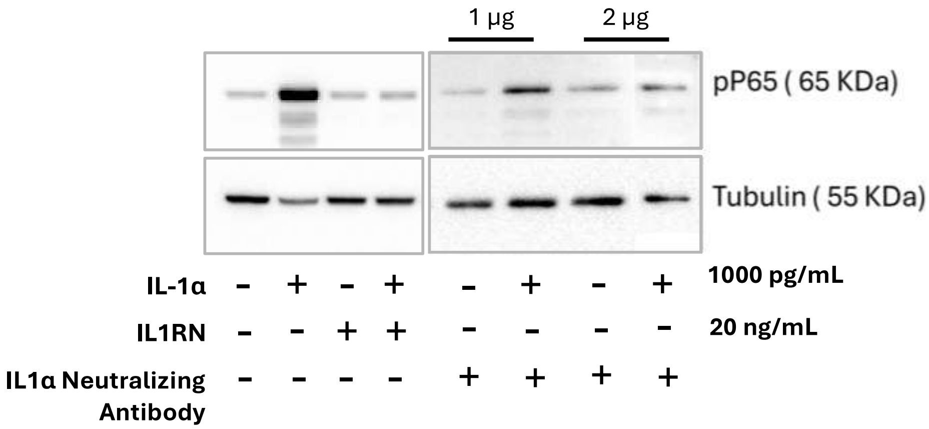

Figure S1: Validation of IL-1α inhibitors. Human primary myocytes were treated with/without IL-1α (10000 pg/mL) for 15 min in the presence/absence of IL1R1 Antagonist (IL1RN, [20 ng/mL]) or IL-1α-Neutralizing antibody 1 µg or 2 µg. Western blots show levels of phospho-p65 (pP65) and Tubulin (loading control).

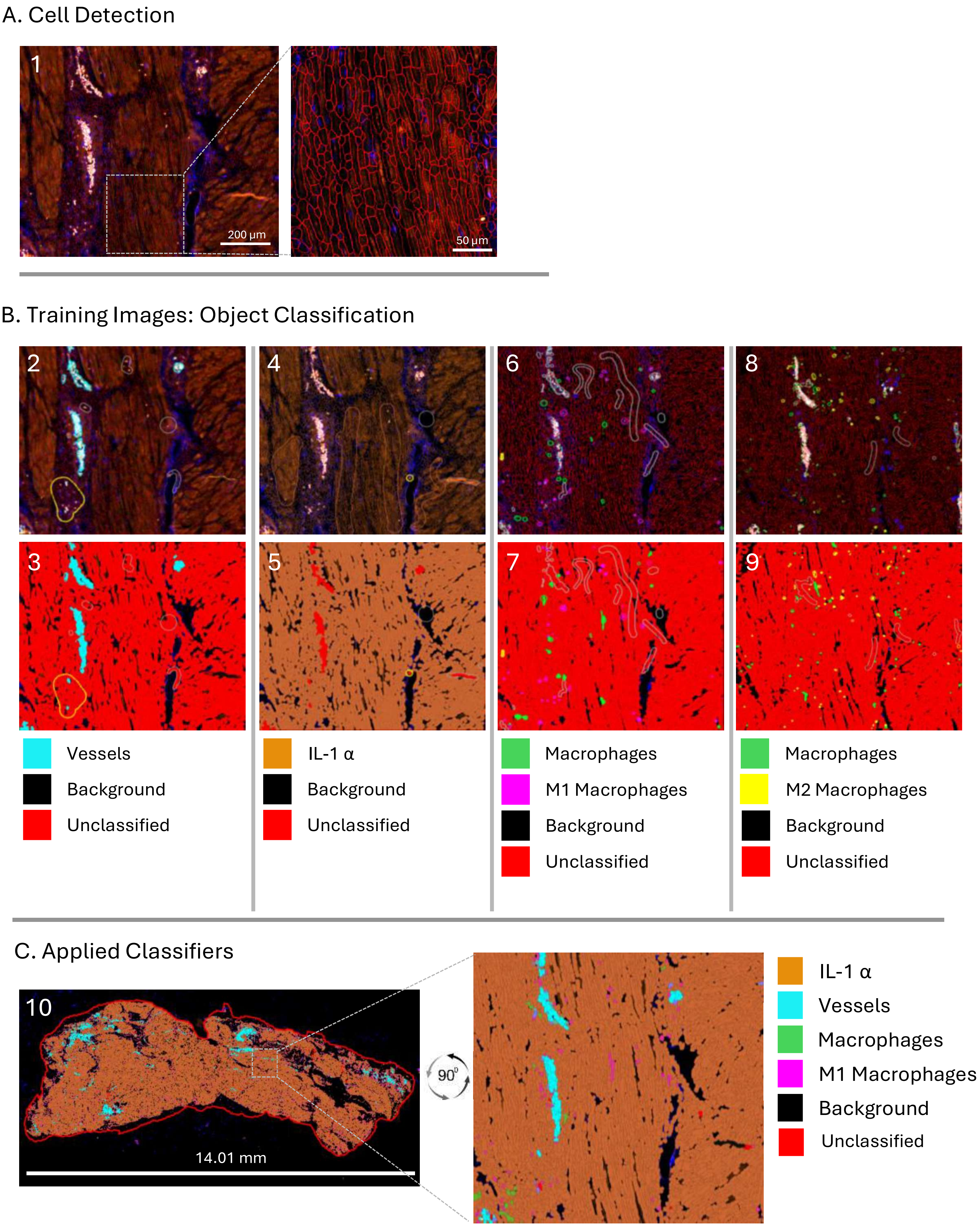

A. Cell Detection
1
200 µm
50 µm
B. Training Images: Object Classification
2
3
Vessels
Background
Unclassified
4
5
IL-1 α
Background
Unclassified
6
7
Macrophages
M1 Macrophages
Background
Unclassified
8
9
Macrophages
M2 Macrophages
Background
Unclassified
C. Applied Classifiers
10
14.01 mm
90°
IL-1 α
Vessels
Macrophages
M1 Macrophages
Background
Unclassified

Figure S2: Detailed workflow of detection and classification of macrophages and myocytes in human myometrium using QuPath analysis. **A)** Cell detection was performed using the channel staining cytoplasm (hence outlining the cells) with parameters: background radius 3 µm, median filter radius 1 µm, and cell expansion 4.79 µm, to segment cells. **B)** Training images for object classification illustrating different objects and cell types: vessels (blue) and background (black) (images 1,2); IL1α-positive cells (brown, includes both myocytes and macrophages) (images 3,4); CD68+iNOS- macrophages (green) and M1 macrophages (magenta) (images 5,6); CD68+ARG-1- macrophages (green) and M2 macrophages (yellow). Upper panel of the image shows training annotations; lower panel shows application of the respective classifier. Unclassified cells are colored red within each training image. C) Example of classifier application across an entire tissue section stained for IL1A, CD68, and iNOS, demonstrating the segmentation and classification outcomes (10). Magnified image has been rotated to 90^0 anti-clockwise.

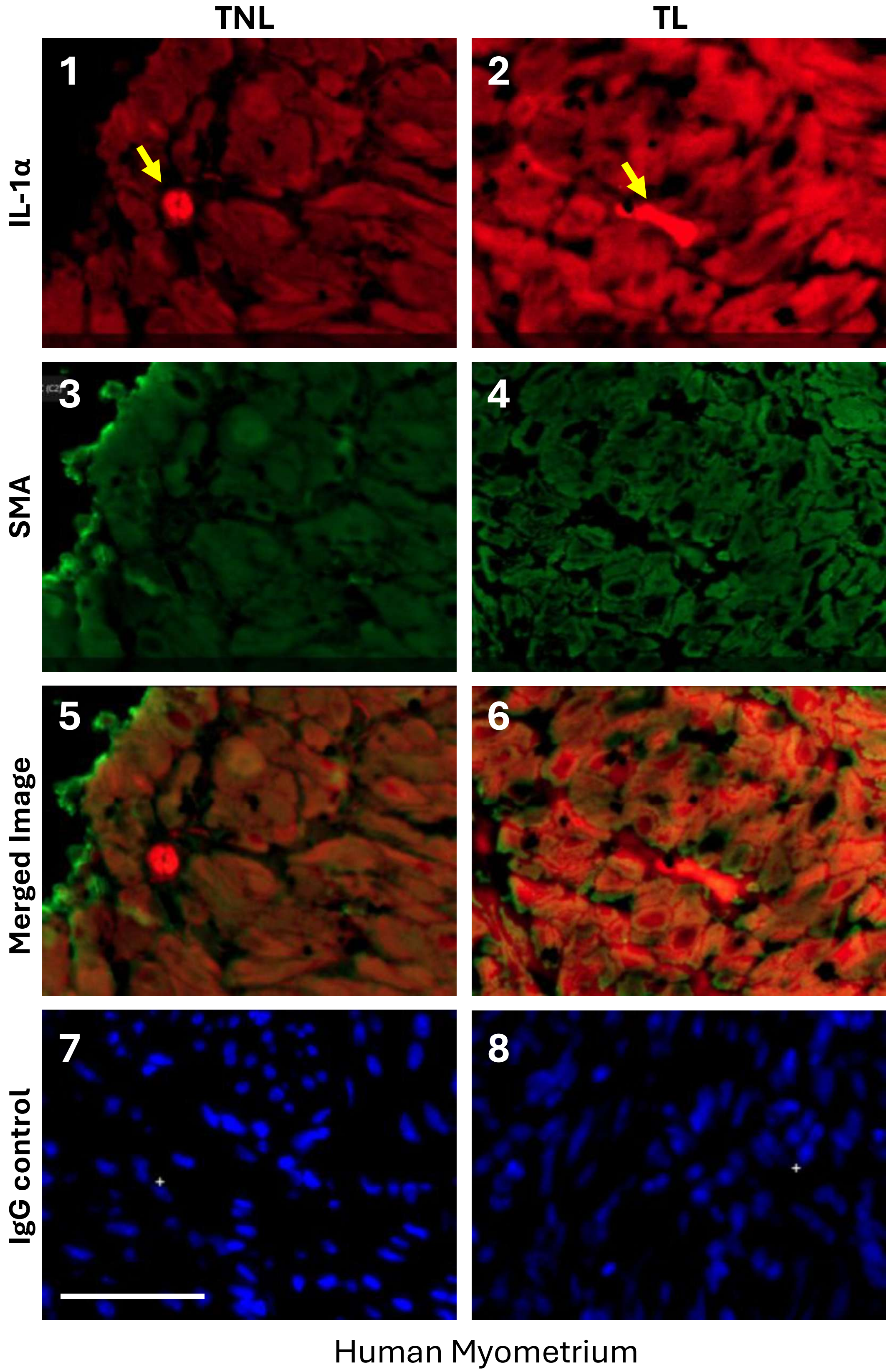

Figure S3: Representative immunofluorescence images showing IL-1α (1-2, Red), Smooth Muscle Actin staining (3-4, green), IgG negative controls (7-8) in myometrial biopsies from women at term not in labour (TNL) and in labour (TL) shown in Figure 1. SMA-ve macrophages are shown with arrows. Scale bar = 50 µm.

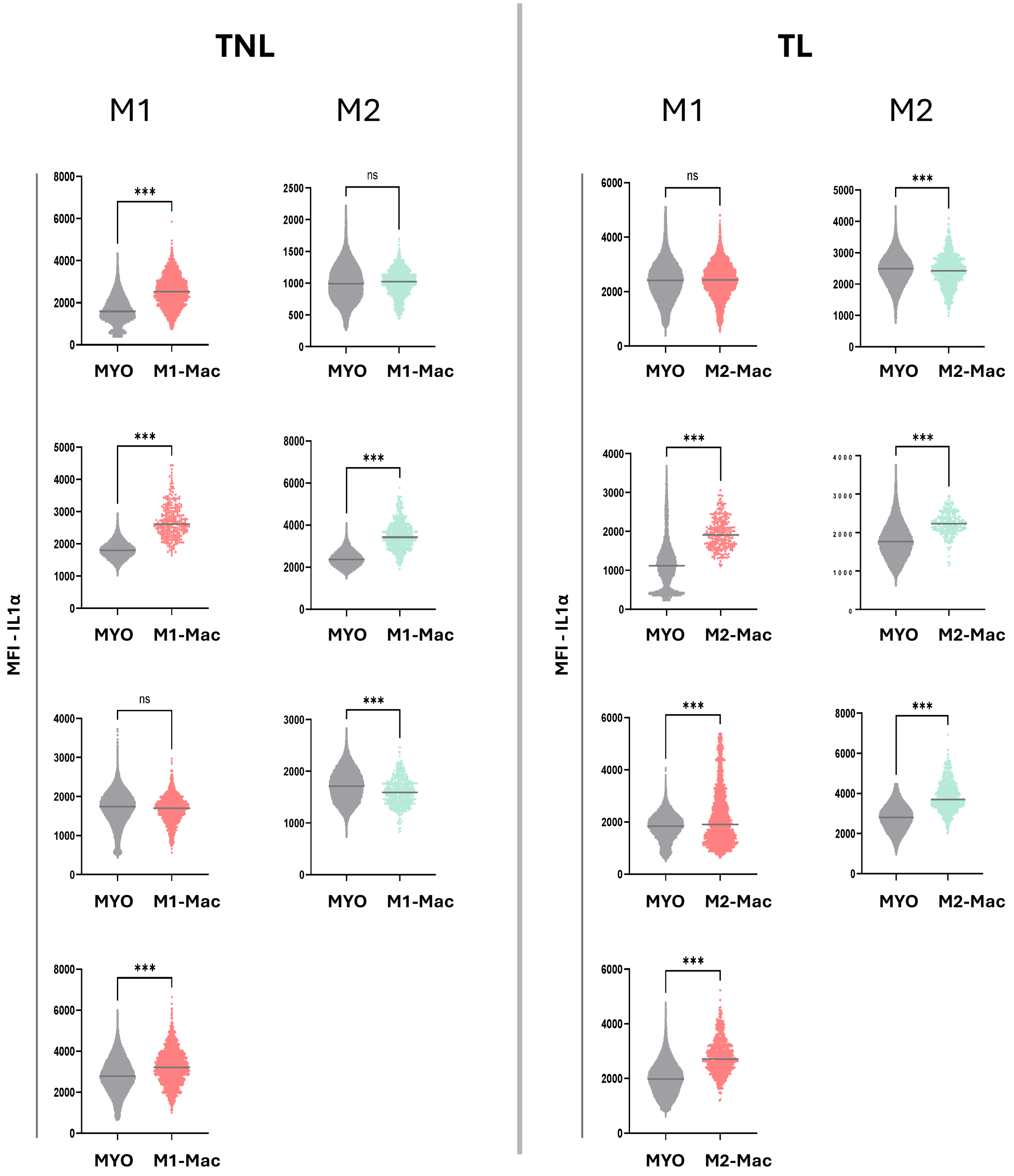

Figure S4: Comparative analysis of IL-1A expression in M1 and M2 macrophages versus myocytes within myometrial biopsies from women with term not in labour (TNL) and term labour (TL), as summarized in Figure 1. Each graph displays the mean fluorescence intensity (MFI) of IL-1A per cell per biopsy, quantified using QuPath software. Scatter plots comprise total population, with each dot representing a cell within its respective group. The number of cells analyzed per tissue sample ranged from 20,000 to 400,000, with biopsy areas ranging from 5 - 50 mm². Statistical significance was determined by unpaired Student's t-test, denoted by'***' = $p \leq 0.001$.

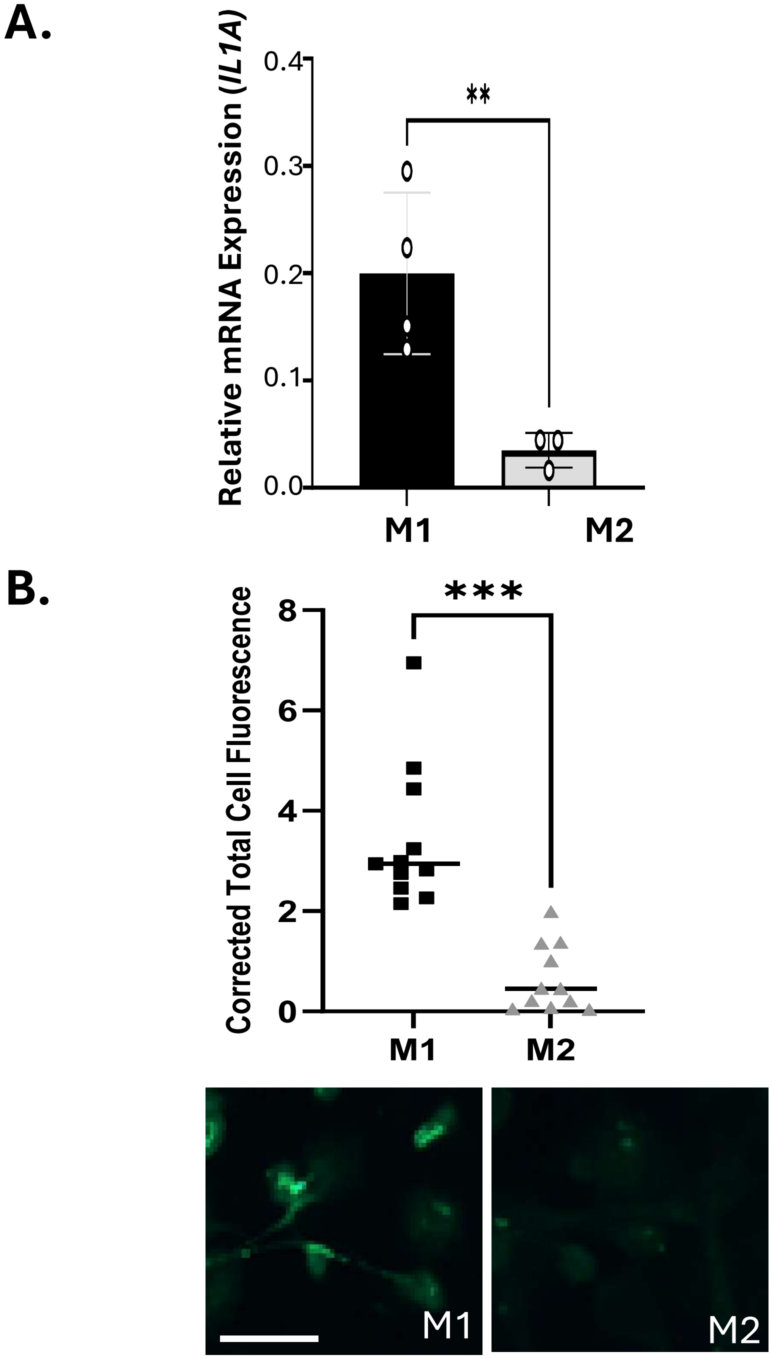

Figure S5: Analysis of IL1α levels in M1 versus M2 macrophages. A) Real time PCR analysis of IL-1α transcript levels in M1 and M2 macrophages, derived from the THP1 monocytic cell line. Graph represents relative mRNA expression normalized to three housekeeping genes. Data are presented as mean ± SD (N=3-4). Statistical significance was determined using unpaired student's t-test denoted by '**' $p \leq 0.01$. **B)** Representative images and quantification of immunofluorescence staining for IL-1α protein in M1 and M2 macrophages derived from the THP1 monocytic cell line. Graph shows Corrected Total Cell Fluorescence determined by CTCF analysis using ImageJ software. Data are pooled from three independent experiments. Statistical significance was determined using unpaired student's t-test and is denoted by '***' = $p \leq 0.001$. Scale bar = 25 μm

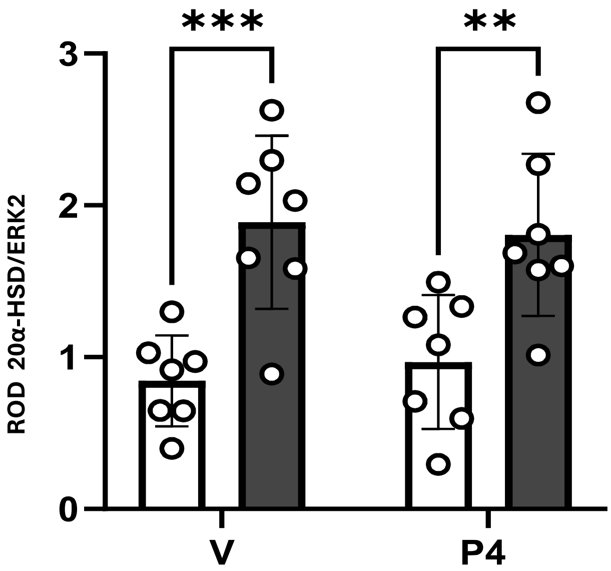

Figure S6: Effect of progesterone on M1-Macrophage induced IL1α in myocytes. Bar graph shows relative quantification of 20α-HSD protein expression in primary human myocytes treated with/without IL-1α (10000 pg/mL) for 24 hours in the presence of progesterone (P4,100 nM) or its vehicle (V). Data are presented as mean ± SD (N=6 term pregnant women) of relative protein levels normalized to ERK2 protein. Statistical significance was determined between Control (white bars) and IL-1α (grey bars) groups and among V and P4 treatments by Two-way ANOVA and Šídák's post test for multiple comparisons; '**' = $p \leq 0.01$; *** = $p \leq 0.001$.

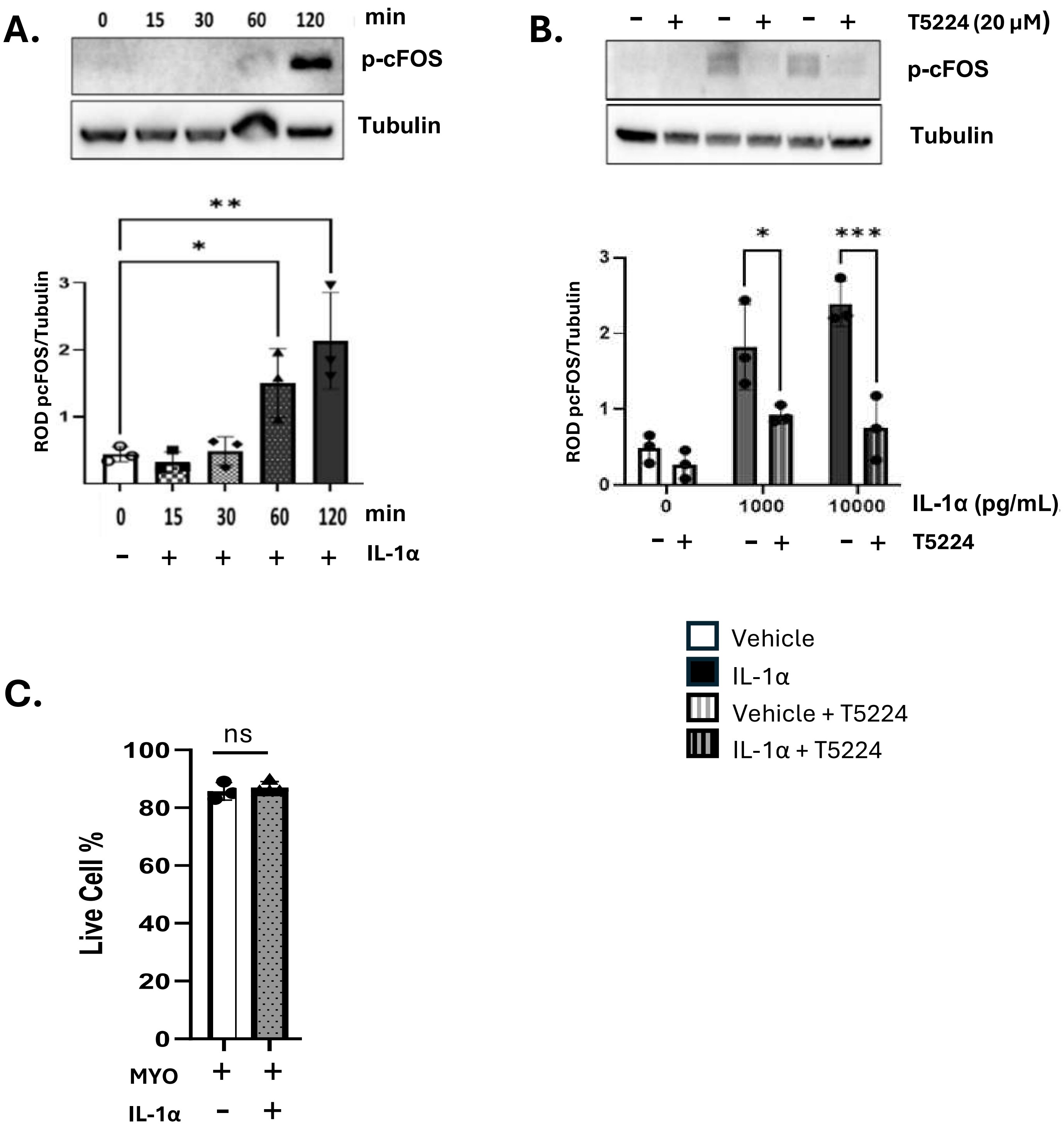
A.
0 15 30 60 120 min
p-cFOS
Tubulin
ROD pcFOS/Tubulin
**
*
0 15 30 60 120 min
- + + + + IL-1α
B.
- + - + - + T5224 (20 μM)
p-cFOS
Tubulin
ROD pcFOS/Tubulin
*
***
0 1000 10000 IL-1α (pg/mL)
- + - + - + T5224
Vehicle
IL-1α
Vehicle + T5224
IL-1α + T5224
C.
ns
Live Cell %
MYO + +
IL-1α - +

Figure S7: Validation of AP-1 inhibition by a specific inhibitor; T5224. A) Representative western blots and densitometric analysis illustrating the induction of phospho-cFOS in myocytes treated with IL-1α (10,000 pg/mL) for 15-120 minutes. **B)** Inhibition of IL-1α-induced p-cFOS in myocytes after pre-treatment with AP-1 inhibitor; T5224 (20 μM) for 30 minutes. Bar graphs show data normalized to Tubulin. Data presented as mean ± SD (N=3). Statistical comparisons were made using One-way ANOVA followed by Dunnett's multiple comparisons **(A)** or Two-way ANOVA with Šídák's multiple comparisons test **(B)**. Statistical significance is denoted by asterisks '*' = $p \leq 0.05$; '**' = $p \leq 0.01$; '***' = $p \leq 0.001$. **C) Cell viability analysis of collagen-embedded myocytes.** Primary human myocytes (Passage 4-6) were released from the collagen gels using collagenase solution (0.1%), washed, centrifuged and re-suspended in Trypan blue solution (0.2%). Cell viability was measured using an automated cell counter. Data represents percent live cells as mean ± SD (N=3). Statistical significance is determined using unpaired student's t-test. 'ns' = non-significant.
